# scSDNE: A semi-supervised method for inferring cell-cell interactions based on graph embedding

**DOI:** 10.1371/journal.pcbi.1013027

**Published:** 2025-05-07

**Authors:** Chenchen Jia, Haiyun Wang, Jianping Zhao, Junfeng Xia, Chunhou Zheng

**Affiliations:** 1 College of Mathematics and System Sciences, Xinjiang University, Urumqi, China; 2 School of Computer Science and Technology, Wuhan University of Science and Technology, Wuhan, China; 3 School of Physical Science and Information Technology, Anhui University, Hefei, China; University of Southern California, UNITED STATES OF AMERICA

## Abstract

As a fundamental characteristic of multicellular organisms, cell-cell communication is achieved through ligand-receptor (L-R) interactions, enabling the exchange of information and revealing the diversity of biological processes and cellular functions. To gain a comprehensive understanding of these complex interaction mechanisms, we constructed a manually curated L-R interaction database and developed a semi-supervised graph embedding model called scSDNE for inferring cell-cell interactions mediated by L-R interactions. scSDNE model utilizes the power of deep learning to map genes from interacting cells into a shared latent space, allowing for a nuanced representation of their relationships. Leveraging the prior information provided by database, scSDNE can infer significant L-R pairs involved in intercellular communication. Experiments on real single-cell RNA sequencing (scRNA-seq) datasets demonstrate that our method detects interactions with a high degree of reliability compared with other methods. More importantly, the model integrates gene regulation information within cells to enhance the accuracy and biological interpretability of the inferences. Our method provides a more comprehensive view of cell-cell interactions, offering new insights into complex intercellular communication.

## Summary

In multicellular organisms, effective communication between cells is essential for coordinating various biological processes, primarily mediated through ligand-receptor interactions. However, most existing methods inadequately consider the impact of intracellular gene regulatory networks, resulting in insufficient capture of global information regarding intercellular interactions and limiting understanding of cellular communication mechanisms. To address this limitation, we propose scSDNE, a semi-supervised graph embedding model designed to infer crucial ligand-receptor pairs that facilitate intercellular communication. By leveraging a manually curated ligand-receptor interaction database and advanced deep learning techniques, scSDNE effectively maps the gene expression data of interacting cells into a shared latent space, thereby enhancing the representation of their relationships. Experimental results demonstrate that scSDNE reliably detects intercellular interactions and improves the biological interpretability of inference results by integrating gene regulatory information. In conclusion, scSDNE can provide valuable insights for complex intercellular communication and understanding disease pathogenesis.

## Introduction

Every cell in multicellular organisms exists within a complex signaling environment [1]. Through intercellular communication and coordination, they collectively accomplish various intricate biological tasks, from early development to tissue and organ maturation [2,3]. Intercellular communication relies extensively on the interaction between ligands expressed by sending cells and homologous receptors expressed by receiving cells [4,5]. Therefore, accurately identifying and analyzing ligand-receptor (L-R) interactions is essential for understanding cell behavior and responses to neighboring cells [1]. Single-cell RNA sequencing (scRNA-seq) is a powerful technique for exploring tissue heterogeneity [6], providing unprecedented resolution to reveal cell diversity and facilitate the exploration of cell-to-cell communication and interactions [7].

Recently, various methods have been developed to infer intercellular communication from scRNA-seq data, such as CellCall [3], CellChat [8], CellPhoneDB [9], NATMI [10], and NicheNet [11]. These methods primarily focus on the expression strength and specificity of L-R pairs. However, this expression-dependent approach has limitations, including the inability to detect certain stable or low-abundance receptor transcripts and the failure to comprehensively reflect changes in intracellular signaling pathways [12]. To utilize information within the receiving cell for constructing communication networks, some methods attempt to integrate insights from L-R interactions and intracellular signaling pathways. For instance, CellCall combines cell-to-cell communication with intracellular transcription factor expression to analyze receptor pathway changes. CINS [13] identifies key ligands and targets involved in interactions between cell types by employing a regression model based on a matrix of ligand-target interactions. Nevertheless, they fail to account for intracellular gene interactions, which may lead to missed biologically significant interactions.

On the other hand, the absence of spatial information in scRNA-seq data restricts its ability to study cell communication in tissues with clear spatial structures [14]. As a result, several approaches have emerged based on spatial transcriptome data. For example, HoloNet [15] infers spatial communication networks across defined regions using a multi-view network approach. Other methods, such as SpaOTsc [16], stLearn [17], and COMMOT [18], integrate scRNA-seq data with spatial imaging data to ascertain the spatial positioning of cells within tissues, thereby inferring cell-cell communication [16–19]. Although spatial transcriptomics offers a new perspective for analyzing cellular communication, its limited resolution and small sample sizes [20] present challenges in thoroughly studying intercellular interactions. In comparison, scRNA-seq is a more mature technique that offers comprehensive information on cell types and gene expression data, facilitating a better understanding of cell-cell interactions and key signaling pathways.

In this context, we developed scSDNE, a semi-supervised graph embedding model designed to infer L-R pairs while incorporating intracellular gene regulatory information. The model takes gene expression data from interacting cells as input and integrates the L-R interaction database (LRdb) to identify significant intercellular communications. scSDNE constructs a weighted adjacency matrix that combines gene regulatory and L-R interaction information. It then employs Structure Deep Network Embedding (SDNE) [21] to capture both first-order and second-order similarities of the network, effectively embedding genes into a shared latent space. By analyzing the distances between L-R genes in the latent space, significant L-R pairs mediating communication between interacting cells can be inferred. Furthermore, experimental results on human atopic dermatitis (AD), gastric cancer, and hepatocellular carcinoma (HCC), demonstrate the model's efficacy in elucidating the overall characteristics of cell communication.

## Results

**Fig 1:**
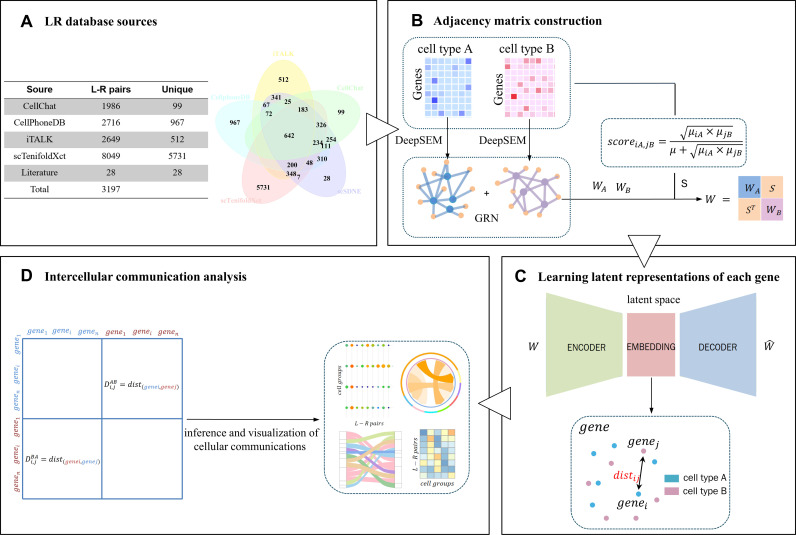
Overview of scSDNE. (A) Overview of the LRdb. (B) Construction of adjacency matrix (including adjacency matrices of gene regulatory networks and crosstalk score matrices of cell types). (C) Learning of latent representations for each gene pair through graph embedding. (D) Detection and visualization of significant L-R pairs based on the distances observed in the latent space.

The core algorithm and the underlying intercellular communication model of scSDNE are depicted in Fig 1. To construct the joint similarity matrix, scSDNE first utilizes a gene expression matrix with annotated cell types to calculate the gene regulatory network adjacency matrix for interacting cell types separately. Thereafter, interaction scores between cell types are computed based on a defined scoring mechanism. This joint similarity matrix W serves as a weighted adjacency matrix input for the graph embedding model SDNE, enabling the extraction of gene coordinate representations within a low-dimensional latent space. Finally, by analyzing the distances between genes in this latent space, significant L-R pairs between interacting cell types are inferred in conjunction with the previously compiled LRdb.

### Inferring cell-cell communications between the human lesional skin cells

AD is a common inflammatory skin disease characterized by a complex pathogenesis involving immune cells and epidermal abnormalities [22]. Previous studies have identified specific skin cell expression of chemokines from fibroblasts to immune cells [23], revealing a potential role in fibroblasts transmitting to immune cells. Therefore, we applied scSDNE to lesional skin scRNA-seq data from AD patients to detect cell communication. The inflammatory skin datasets encompasses twelve distinct cell sub-types, including four fibroblast sub-types (APOE + FIB, FBN1 + FIB, COL11A + FIB, and Inflam. FIB), four dendritic cell sub-types (cDC1, cDC2, LC and Inflam. DC), as well as four T cell sub-types (TC, Inflam. TC, CD40LG + TC and NKT). Given that the hallmark of AD lesions involves unique interactions between Inflam. FIB and immune cells [22], we focused on analyzing Inflam. FIB as sending cells and their interactions with other immune cells.

**Fig 2:**
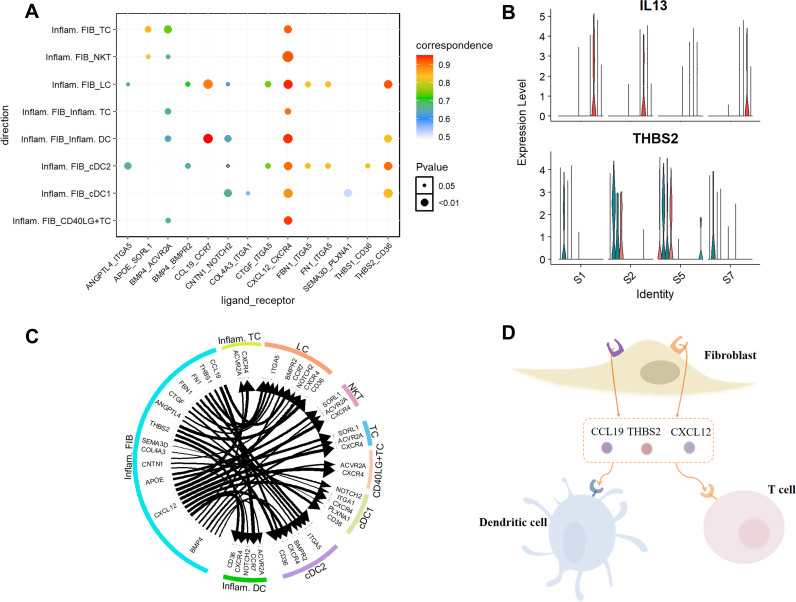
Identification of intercellular communication in diseased human skin. **(A)** Dot plot displays the predicted interactions between Inflam. FIB and the specified immune cell types. The color of the points reflects the communication probability, while the size of the points represents the calculated p-value. Blank areas signify a communication probability of zero. **(B)** Violin plot shows the expression levels of genes IL-13 and THBS2 across samples. **(C)** Circos plot depicts intercellular communication from Inflam. FIB to other cell types. The arrow points from the ligands in the sending cells to the receptors in the receiving cells. The thickness of the line and the size of the arrow reflect the expression of the ligands and receptors, respectively. **(D)** Illustration of representative ligands from Inflam. FIB to other cell types.

The interactions identified when Inflam. FIB act as sending cells concentrate on 3 key ligand genes: CCL19, CXCL12, and THBS2 (Fig 2A, 2C, and 2D). Specifically, CCL19 plays a crucial role in lymphocyte recirculation, homing, and migration to secondary lymphoid organs. The significant interaction between CCL19 and its receptor CCR7 highlights the regulatory effects of Inflam. FIB on lymphocytes and modulates type 2 inflammation involving Th2 cells, a CD4 + T cell subset associated with inflammation [24]. Furthermore, CXCL12 and its receptor CXCR4 exhibit high activity in lesional skin. Notably, a study by Sun et al. [25] observed that in AD, CXCL12 acts as a chemokine to recruit inflammatory cells, facilitating their migration regions by binding to CXCR4, thereby activating these cells to release inflammatory cytokines and accelerating the progression of inflammation [26].

By examining the roles of ligands THBS2 and THBS1, we found that they effectively inhibit angiogenesis through their interaction with the receptor CD36 [27, 28]. Moreover, interleukin-13 (IL-13) is closely associated with disease severity [29] and demonstrates a positive correlation with inflammatory processes. In Fig 2B, the expression levels of IL-13 are significantly lower in the third sample compared to the other samples, while expression of THBS2 is markedly elevated in this sample. This finding suggests a potential relationship between the down-regulation of IL-13 and the up-regulation of THBS2 expression under specific conditions, subsequently influencing the inflammatory processes and angiogenesis associated with AD. Therefore, further exploration of the interaction mechanism between IL-13 and THBS2 is essential for enhancing our understanding of the pathophysiological processes in AD and for the development of novel therapeutic strategies.

### Revealing cellular crosstalk in tumor-adjacent tissues and HCC tissues

**Fig 3:**
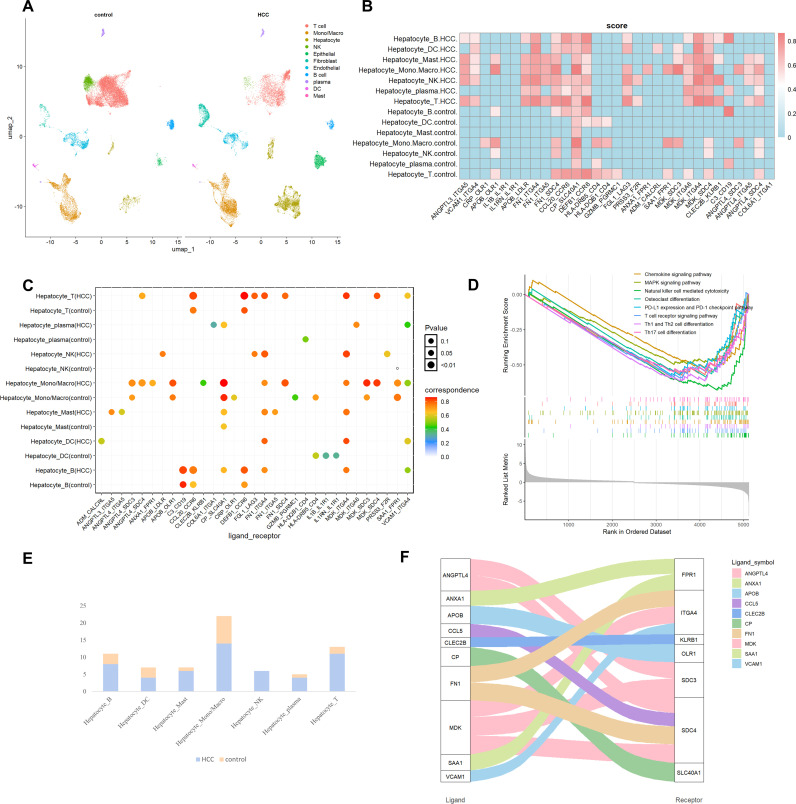
Comparison analysis of intercellular communication between HCC and tumor-adjacent tissues. **(A)** Overview of the cell clusters derived from scRNA-seq data of tumor-adjacent and HCC tissues (UMAP). **(B)** Heatmap illustrates intercellular communication from hepatocytes to other immune cell types (normalized score greater than 0. 5). **(C)** Dot plot shows the predicted interactions between hepatocyte and the specified immune cell types in both HCC and tumor-adjacent tissues. **(D)** Enrichment analysis of the KEGG pathways. **(E)** Comparison of the number of significant L-R pairs from hepatocytes to immune cell types. **(F)** Sankey plot represents intercellular communication from hepatocytes to Mono/Macro in HCC where the thickness of the connecting bands reflects the intensity of L-R interactions.

HCC, one of the most malignant cancers, exhibits a highly heterogeneous complex ecosystem characterized by complex intercellular communication between different cell types [30]. After processing data from tumor-adjacent and primary tumor tissues of 5 HCC patients in GSE149614, we identified a total of 8 cell types: T cells, B cells, NK cells, plasma cells, hepatocytes (malignant cells in HCC), dendritic cells (DC), Mono/Macro, and mast cells (Fig 3A) [31,32].

Upon further analysis, we noted a significant reduction in the proportion of T cells and NK cells within tumor tissues decreased compared to adjacent tissues, whereas Mono/Macro and malignant cells constituted a higher proportion of the immune component in the primary tumor. This finding reveals that as HCC progresses, plenty of immune cells are recruited to the liver and interact closely with stromal cells to jointly construct an active immune micro-environment, which is of great significance for the occurrence and development of liver cancer [31]. To delve into mechanisms of interaction between the liver and immune cells, we selected highly variable genes and applied the scSDNE for in-depth analysis.

Intercellular interactions mediated by ligands FN1 and MDK, along with their receptors ITGA4, ITGA5, and ITGA6, have been extensively documented in HCC tissues, but rarely identified in normal tissues (Fig 3B and 3C). As a critical glycoprotein, FN1 has not only been confirmed in previous studies to promote metastasis, angiogenesis and proliferation of cancer cells, but also can bind to ITGA5 and ITGB1 to trigger the recruitment and activation of signaling pathway-related proteins, including FAK/Src complex, which exerts a profound impact on tumor progression [33–35]. It has been reported that MDK interacts with the ITGA4 and ITGA6 receptors, activating a series of downstream signaling cascades that significantly enhance cancer cell growth, migration, metastasis, and angiogenesis, thereby further exacerbating the progression of HCC [36]. Notably, FGL1-LAG3 is highly expressed in HCC, particularly in T and NK cells. Multiple studies have indicated that FGL1 binding to LAG3 impairs T cell function and promotes immune escape, thereby suppressing immune responses [37–39]. This observation aligns with Gene Set Enrichment Analysis (GSEA) results suggesting the down-regulation of T/NK cell-related immune pathways in HCC, underscoring the critical role of FGL1-LAG3 in immune evasion in HCC (Fig 3D).

As illustrated in Fig 3E, there is extensive intercellular communication between hepatocytes/malignant cells and immune cells, especially Mono/Macro, with this interaction being markedly more pronounced in HCC. This phenomenon prompts us to further investigate the interaction between malignant cells and Mono/Macro within tumor tissues. Fig 3F indicates that malignant cells primarily communicate with Mono/Macro through receptor SDC4, particularly involving L-R pairs such as ANGPTL4-SDC4, FD1-SDC4, and MDK-SDC4, which are absent in adjacent tumor tissues. This suggests that SDC4, as a key endogenous membrane receptor, may exert a crucial role in tumor initiation and development by modulating cellular adhesion and migration in various cancers through its interactions with ligands [40–42]. Additionally, scSDNE successfully detected that receptor ITGA4 in Mono/Macro forms another important set of L-R pairs with the ligands FN1, MDK, VCAM1 expressed by malignant cells (Fig 3F). Notably, VCAM1 is strongly associated with malignant tumor development, as confirmed by multiple studies [43,44]. These observations further underscore the accuracy of scSDNE.

### Analysis of cell communication in the human lymph node microenvironment

**Fig 4:**
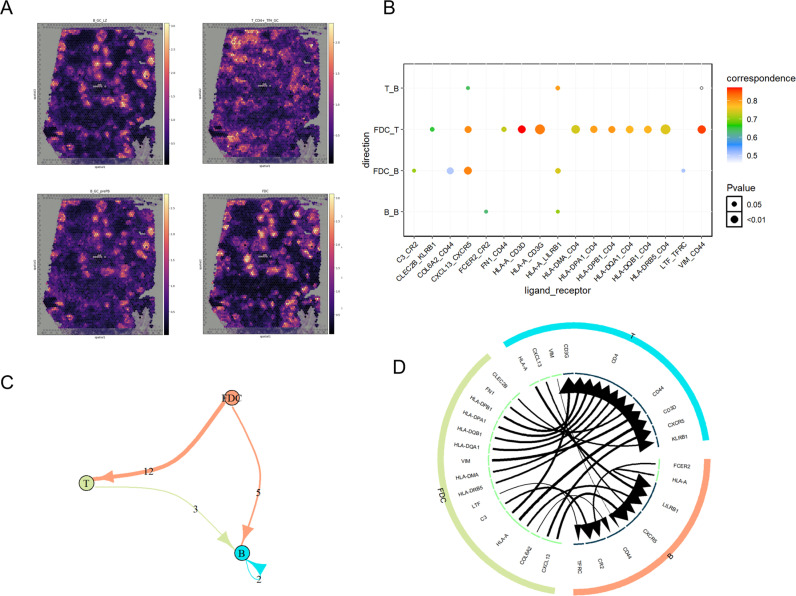
A case study on the application of scSDNE in the human lymph node microenvironment. **(A)** Spatial plots show cell abundance (color intensity) for the specified cell types. **(B)** Dot plot shows the predicted interactions between the immune cell types. **(C)** The edge width represents the strength of intercellular communication between the cell types. **(D)** Circos plot. The arrow points from the ligands in the sending cells to the receptors in the receiving cells.

The human lymph node is characterized by a dynamic micro-environment with many spatially intermingled cell populations [45]. We used cell2location to integrate the Visium human lymph node datasets from 10x Genomics with the scRNA-seq datasets from human secondary lymphoid organs. According to the analysis by cell2location, in the germinal center (GC) light zone, B cells are selected by T follicular helper (Tfh) cells and follicular dendritic cells (FDCs) to differentiate into antibody-producing plasma cells [45]. Therefore, we chose to study B cells, T cells, and FDCs enriched in the GCs to investigate the cell communication during the B cell differentiation process (Fig 4A).

The results show that the number of L-R interactions between FDCs and T cells is most significant, with the majority mediated by genes encoding MHC II molecules (Fig 4B and 4D). The study indicated that There are significant interactions between human MHC II molecules encoded by the HLA-DP, HLA-DQ, and HLA-DR genes on FDCs and T cells [46]. CD4 + T cells can bind to antigens on MHC class II molecules, thereby activating B cells, macrophages, and other T cells [47,48]. This process not only enhances the activity of other immune cells but also promotes the development of memory T cells and B cells.

Additionally, the interaction between CXCL13 and CXCR5 among cell types in this region was generally significant (Fig 4B and 4C). The study demonstrated that CXCL13 plays a crucial role in coordinating cell migration within different regions of secondary lymphoid organs [49], primarily through its receptor CXCR5. CXCL13-CXCR5 guides B cell migration to lymphoid follicles, promotes the formation of lymphoid follicles, and participates in B cell and T cell-mediated immune responses. In the light zone, B cells from the dark zone can acquire antigens from FDCs, assisting in the formation of Tfh cells, and selectively re-enter the dark zone or exit the germinal center by differentiating into long-lived plasma cells and memory B cells [49, 50].

### Application of scSDNE in gastric cancer TME and comparative analysis with various methods

The tumor micro-environment (TME) in gastric cancer plays a critical role in regulating tumor progression through complex cellular interactions [51,52]. Therefore, we applied scSDNE to analyze the scRNA-seq datasets of gastric cancer. After processing the superficial and deep tumor invasion data from five patients in GSE167297, a total of 10 different cell types were identified (S1 and S2 Figs). Cancer-associated fibroblasts (CAFs) are a crucial component of the TME in gastric cancer, contributing significantly to the recruitment of immune-suppressive cells and facilitating immune evasion [53–55]. Therefore, our analysis primarily focuses on the interactions of fibroblasts, which serve as sender cells, with other cell types within the TME.

scSDNE detected 76 L-R pairs between fibroblasts and immune cells (S3 Fig). Among these, collagen family genes and FN1 highly expressed in CAFs interact with the CD44 receptor on immune cells, driving cancer progression [56–59]. Additionally, laminin encoded by LAMA4, LAMB1, and LAMB2 mediate adhesion through Integrin binding—specifically the Integrin α6β4, composed of integrin α6 (ITGA6) and Integrin β4 (ITGB4)—is suggested to be essential for tumor development and progression, promoting pro-cancer signaling pathways [60].

**Fig 5:**
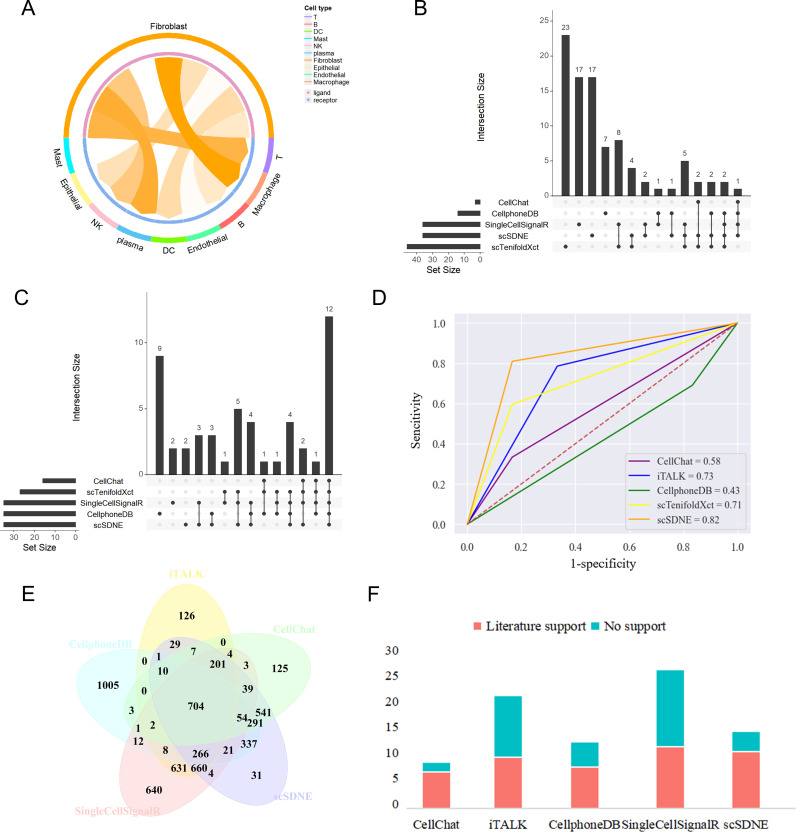
Comparison of the performance of scSDNE with CellChat, CellPhoneDB, iTALK and scTenifoldXct on the scRNA-seq dataset of gastric cancer (from fibroblasts to macrophages). **(A)** Circos plot depicts the strength of intercellular communication from fibroblasts to other cell types in TME. **(B)** UpSetR plot illustrates the results from the five tools utilizing their respective LR database. The horizontal bar graph in the lower left corner represents the total number of L-R pairs detected by each method. The intercellular communication results obtained by the different methods are represented by multiple black dots and connecting lines, and the number of intersections displayed in the bar graph above. **(C)** UpSetR plot shows the results from the five tools using a common LRdb. **(D)** ROC curves plot depicts the performance of the five methods in assessing intercellular communication. **(E)** Overlap analysis of the LR database. **(F)** Comparison of literature support rates for scSDNE using different databases.

As shown in Fig 5A, macrophages serve as the primary recipients of signals from fibroblasts. We compared the accuracy and effectiveness of scSDNE with several established methods (CellChat, CellPhoneDB, iTALK [61], and scTenifoldXct) on these two cell types. As shown in Fig 5B, using their respective databases, scSDNE identified 14 unique communications that were not detected by any other method, while CellPhoneDB, iTALK and scTenifoldXct identified 6, 17, and 23 particular communications; CellChat did not identify any unique communications. Significantly, the predictions from scTenifoldXct displayed considerable divergence from those of other methods, likely due to the reduced overlap of its L-R database with others, thereby highlighting the critical impact of the selected database on prediction outcomes (S4 Fig).

To facilitate a fair comparison of the predictive performance across these methods, we utilized a unified LR database as a benchmark for further analysis. scSDNE, CellChat, CellPhoneDB, iTALK and scTenifoldXct identified 35, 15, 35, 34, and 26 intercellular communications from fibroblasts to macrophages, respectively. Fig 5C demonstrates that when all methods utilized the same database, the consistency of the detected L-R pairs significantly improved. Among the methods, CellChat and scTenifoldXct identified fewer L-R pairs compared to others and did not reveal any specific cell-cell communications. Importantly, scSDNE not only demonstrated superiority in the number of identified L-R pairs, but also showed a considerable overlap in detection results with those of other methods.

Furthermore, using relevant literature reports as evaluation criterion, we compared the predictive performance of these methods using ROC curves (Fig 5D). The results indicated that scSDNE achieved the highest area under the ROC curve (AUC = 0. 82), indicating its comprehensiveness and accuracy in capturing cell-cell interactions.

To further assess effectiveness, we utilized scSDNE in conjunction with five different databases (scSDNE, CellChat, CellPhoneDB, iTALK, and SingleCellSignalR) to analyze the interactions of fibroblasts with Mono/Macro in HCC. Fig 5E shows that there are overlapping regions among these five databases. When using different databases, the number of literature-supported L-R pairs detected by scSDNE was similar, indicating that its predictive results have high stability and consistency (Fig 5F).

## Discussion

In fact, GRNs encompass most known and unknown gene regulatory information, including L-R secretion and the activities of downstream regulatory factors [62]. To this end, we introduced scSDNE, a model that integrates intercellular L-R interactions with regulatory information among intracellular genes to infer and analyze cell-cell communication. scSDNE embeds genes into a shared latent space, calculating the distances between all gene pairs, which allows for the identification of L-R pairs that may have been overlooked by other methods but are nonetheless of significant investigative relevance. Importantly, given that most L-R pairs in LRdb overlap with other databases, LRdb is regarded as a reliable resource.

Case studies on scRNA-seq data from human lesional AD skin, HCC and gastric cancer demonstrate that scSDNE effectively infers intercellular communication, exhibiting superior accuracy compared to other tools. A case study focusing on lesional AD skin revealed notable signal crosstalk among immune cells. Many L-R pairs inferred from Inflam. FIB, such as CXCL12-CXCR4 and CCL19-CCR7, have been confirmed in multiple studies as primarily mediators of cell recruitment to inflammatory regions, significantly promoting inflammation exacerbation and development [24–26]. Additionally, we identified that the THBS2-CD36 interaction may influence the pathological process of AD through the inhibition of angiogenesis, meriting further investigation.

Analysis of the HCC datasets unveiled a complex interaction network between hepatocytes and immune cells, highlighting the key roles of FN1, MDK, and their receptors ITGA4, ITGA5, ITGA6. This analysis revealed significant L-R interactions between malignant cells and Mono/Macro, with the receptor SDC4 playing a crucial role. Furthermore, we observed notable metabolic differences between tumor tissues and adjacent tissues with significant interactions involving FGL1-LAG3 being negatively correlated with T cell subset numbers, indicating down-regulation of immune pathways and highlighting their immunosuppressive effects in liver cancer [37–39].

cell2location combines Visium and scRNA-seq data, allowing scSDNE to deeply analyze intercellular communication within the human lymph node micro-environment. The study reveals key interaction mechanisms between germinal center B cells, T cells, and FDCs. The results indicate that the MHC class II molecule-mediated interactions between FDCs and T cells are the most significant, promoting the activation of B cells, macrophages, and T cells. Furthermore, the CXCL13-CXCR5 signaling pathway plays a crucial role in regulating B cell migration and lymph follicle formation, further supporting the collaborative function of immune cells [49,50].

Through scSDNE analysis, we also elucidated L-R interactions between fibroblasts and immune cells—particularly macrophages—in gastric cancer, where interactions of CD44 with collagen family genes and FN1 emerged as key mechanisms for information exchange. The substantial enrichment of multiple signaling pathways further underscores the significance of these key L-R pairs in the pathological mechanisms of gastric cancer.

scSDNE represents a semi-supervised graph embedding approach designed to infer intercellular communication. By effectively leveraging gene expression regulation information derived from scRNA-seq data, it accurately identifies L-R pairs that are biologically significant between interacting cells. Although scSDNE performs excellently in inferring intercellular communication, its requirement for users to manually select sending and receiving cells somewhat limits its applicability. To overcome this limitation, future research will focus on developing automated algorithms to reduce reliance on user intervention, thereby enhancing the method's applicability and efficiency in complex biological environments. Additionally, integrating spatial transcriptomics data to obtain micro-environment information will enable scSDNE to predict intercellular interactions at the spatial tissue level. In summary, scSDNE, as an effective tool, has broad application prospects for analyzing intercellular communication at the single-cell level, and its future development will open new possibilities for biomedical research.

## Methods

### Data processing

The input of scSDNE is a gene expression matrix with annotated cell types. In scRNA-seq datasets, low-quality cells are filtered out based on metrics such as gene counts and the ratios of RNA and mitochondrial genes per cell. The data is then normalized using the NormalizeData function from Seurat [63]. To integrate data from different samples, batch effect correction is performed using the R package “Harmony” to address technical differences between samples. Subsequently, PCA and UMAP [64] are employed for data dimensional reduction and visualization. Cell type annotation is accomplished by identifying marker genes for each cell cluster. Finally, the analysis focuses on highly variable genes to highlight gene features that are most representative of distinct cell states.

### Crosstalk score calculation

Assume that gene i has an average expression level of $u_{iA}$ in cell type A, and gene j has an average expression level of $u_{jB}$ in cell type B. According to the scoring method described in SingleCellSignalR [65], the interaction score between gene i in cell type A and gene j in cell type B is denoted as follows:


$${score}_{iA,jB}=\frac{\sqrt{u_{iA}u_{jB}}}{c+\sqrt{u_{iA}u_{jB}}}$$ (1)

where c=mean(C) and C is the count matrix for cells A and B. Hence, the interaction score matrix $S\in {\mathbb{R}}^{n\times n}$ consists of ${score}_{iA,jB}$ between cell types A and B, with all elements in S ranging from [0, 1).

### Gene regulatory network construction

Given the gene expression matrices $X_{A}$ and $X_{B}$ for two cell types, A and B, DeepSEM [66] can infer the gene regulatory networks (GRNs) within each cell type. DeepSEM is a deep generative model that jointly infers GRNs and the biological representation of single-cell RNA sequencing data. Its framework is built on a beta-variational auto-encoder (beta-VAE), where the weights of the encoder and decoder functions directly represent the GRN adjacency matrix. $W_{A}$ and $W_{B}$ represent GRN adjacency matrices, with the absolute value of an element indicating the likelihood that gene i regulates gene j. Since the GRN constructed by DeepSEM is a directed graph, we require the likelihood of a regulatory relationship between two genes, necessitating that the constructed matrix W be symmetric and each element non-negative. To ensure symmetric, we define $W_{ij}=W_{ji}=max(W_{ij},W_{ji})$.

Given the significant differences between the edge weights of the GRN and the interaction scores, a scaling factor μ is introduced to ensure that the regulatory information of genes and the crosstalk scores are comparable. This adjustment allows their contributions in the combined similarity matrix X to be roughly equal, preventing bias towards one measure during the embedding process. Referencing scTenifoldXct [67], the scaling factor μ is set as follows:


$$\mu =\frac{\sum_{i,j}{\left(W_{A}\right)}_{ij}+\sum_{i,j}{\left(W_{B}\right)}_{ij}}{2\sum_{i,j}{score}_{iA,jB}}$$ (2)

Since matrices $W_{A}$ and $W_{B}$ are relatively sparse, it is essential to identify the positions of the non-zero elements in both $W_{A}$ and $W_{B}$ to utilize them more effectively. Then, the corresponding elements in the crosstalk score matrix S are summed to obtain s. Consequently, let


$$\mu =\frac{\sum_{i,j}{\left(W_{A}\right)}_{ij}+\sum_{i,j}{\left(W_{B}\right)}_{ij}}{2s},\;W_{A}^{\;\prime }\;=\;\frac{W_{A}}{\mu },\;W_{B}^{\;\prime }\;=\;\frac{W_{B}}{\mu },$$

and X construct the joint similarity matrix as follows:


$$X=[\begin{array}{cc} W_{A}^{\;\prime } & S\\ {}[2pt]S^{T} & W_{B}^{\;\prime } \end{array}].$$

### Learning latent representations of each gene

Using the constructed weighted adjacency matrix X as input, the SDNE is employed to reconstruct and obtain the coordinates of genes from both the sender and receiver cells within a unified latent space. SDNE is a semi-supervised deep learning model that simultaneously optimizes both first- order and second-order similarities. First-order similarity refers to the local similarity between pairs of vertices, where the similarity is proportional to the edge weights and serves as supervised information to capture the local structure of the network. In contrast, second-order similarity describes the relationships between vertices and their neighborhoods, providing unsupervised information to capture the global structure of the network.

The loss function of scSDNE can be expressed as follows:


$${ℒ}_{mix}={ℒ}_{2nd}+{ℒ}_{1st}+{ℒ}_{reg}={\left\|\hat{X}-X\right\|}_{2}^{2}+2tr\left(Y^{T}LY\right)+\frac{1}{2}\underset{k=1}{\sum^{K}}\left({\left\|W^{\left(K\right)}\right\|}_{F}^{2}+{\left\|{\hat{W}}^{\left(K\right)}\right\|}_{F}^{2}\right)$$ (3)

where ${ℒ}_{reg}$ represents the regularization term to prevent over-fitting, and $W^{\left(K\right)}$, ${\hat{W}}^{\left(K\right)}$ denote the weight matrices of the K-th layer of the auto-encoder. L is the Laplacian matrix, where L=D−X, $D\in {\mathbb{R}}^{n\times n}$ are diagonal matrices with diagonal elements $D_{i,i}=\sum_{j}\;X_{i,j}$. To avoid mapping all instances to zero, an additional constraint $Y^{T}DY=I_{n}$ is added, with $I_{n}\in {\mathbb{R}}^{n\times n}$ being an identity matrix. To satisfy these constraints, the optimization method proposed by Nguyen et al. [68] is employed. This method computes Riemannian gradients by projecting the gradient onto the tangent space of the Stiefel manifold. These gradients are then employed to update the parameters, ensuring that the output of the optimization problem remains constrained within Stiefel manifold.

The SDNE model consists of an encoder and a decoder, each featuring multiple stacked linear layers, with a Sigmoid activation function applied after each layer to facilitate the network's learning of non-linear data mappings. The encoder transforms the adjacency matrix, which contains interaction scores between cell types and gene regulatory relationships, into a hidden representation. Subsequently, the decoder reconstructs this representation to yield the output adjacency matrix. By minimizing the loss function, scSDNE effectively captures the local structural information of nodes, particularly emphasizing multi-order neighborhood relationships.

### Determining the statistical significance of interactions between cell types

The obtained $Y=[\begin{array}{c} Y_{A}\\ Y_{B} \end{array}]\in {\mathbb{R}}^{2n\times 2n}$ by minimizing the loss function represents the embedding of all genes in a low-dimensional latent space. Where $Y_{A}$ and $Y_{B}\in {\mathbb{R}}^{n\times n}$ are low-dimensional representations of genes in cell types A and B, respectively. Non-parametric tests are then used to determine the statistical significance of L-R pairs. Under the null hypothesis that there are no LR-mediated interactions between gene pairs, important gene pairs among all combinations are identified. The Euclidean distance across cell types for each paired gene in cell types A and B is calculated. After collecting the distances of gene pairs excluding L-R in the LRdb, the null hypothesis distribution ${dist}_{null}$ can be obtained. Gene pairs that are not present in the database but appear in the combinations are considered less likely to be LR-mediated interacting gene pairs. Next, the percentiles of L-R pairs under the null hypothesis distribution are calculated as follows:


$$p-value=\frac{percentile({dist}_{null},{\;dist}_{i})}{100}$$ (4)

and set it as the original p-value. The threshold for all datasets is set at 0.05.

## Supporting information

S1 Fig UMAP plot of scRNA-seq data. UMAP plot shows ten cell types from 19865 cells across 5 patients in the GSE167297 datasets.
(TIF)

S2 Fig Clustering heatmap. Heatmap depicts the expression levels of marker gene in indicated cell types, with cell types displayed at the top and corresponding marker genes listed at the bottom.
(TIF)

S3 Fig Dot plot of cell-cell interactions. Dot plot shows the predicted interactions between fibroblasts and other cell types.
(TIF)

S4 Fig Wayne diagram of the 5 L-R pairs database. Overlap analysis of the LR database.
(TIF)
