# Supplementary figures and images for: scSDNE: A semi-supervised method for inferring cell-cell interactions based on graph embedding
Source: PLoS Comput Biol. 2025 May 7;21(5):e1013027. doi: 10.1371/journal.pcbi.1013027 (PMC12072665; doi:10.1371/journal.pcbi.1013027)

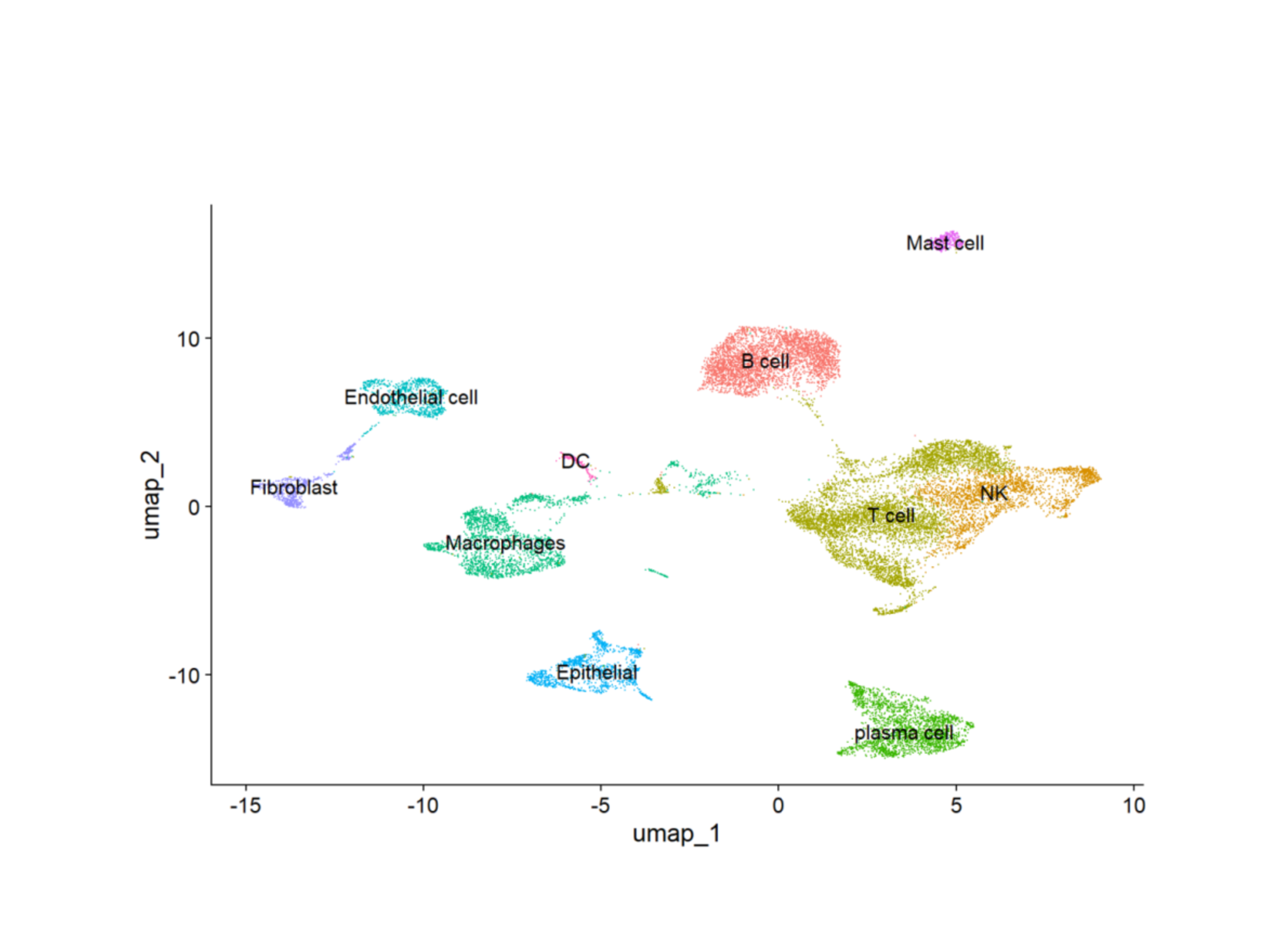

Supplement: S1 Fig — UMAP plot shows ten cell types from 19865 cells across 5 patients in the GSE167297 datasets. (TIF) [file pcbi.1013027.s001.tif]

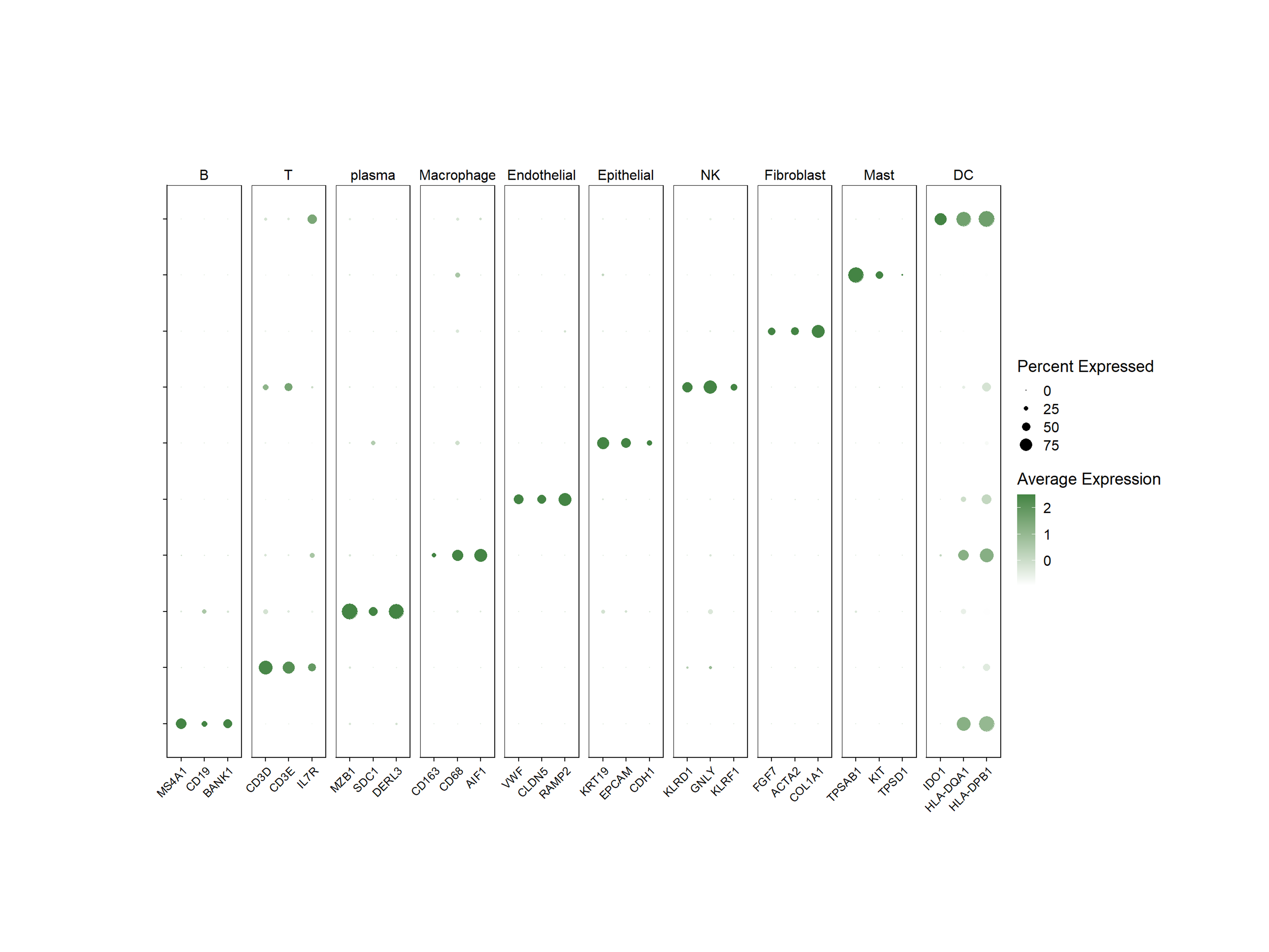

Supplement: S2 Fig — Heatmap depicts the expression levels of marker gene in indicated cell types, with cell types displayed at the top and corresponding marker genes listed at the bottom. (TIF) [file pcbi.1013027.s002.tif]

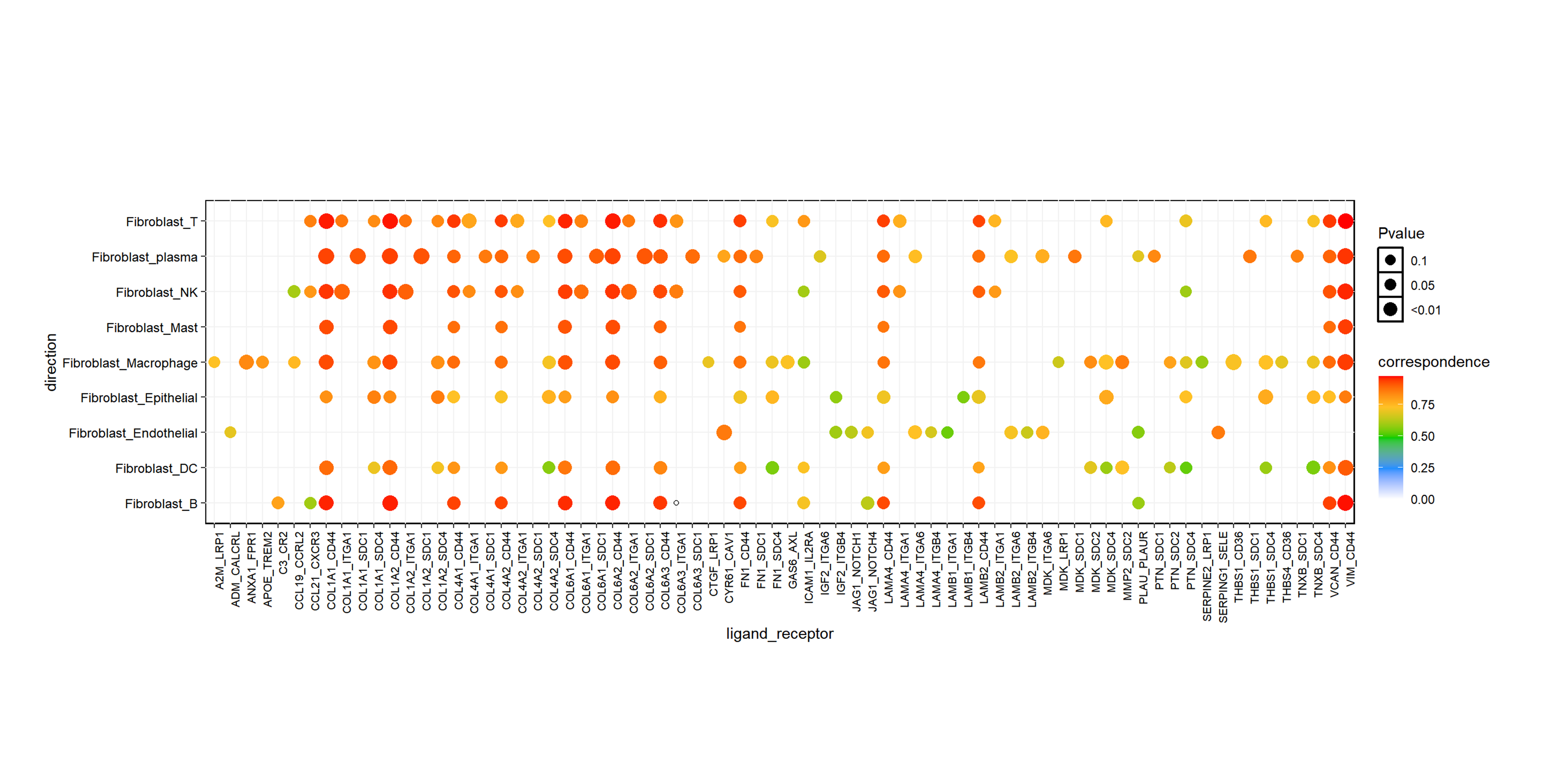

Supplement: S3 Fig — Dot plot shows the predicted interactions between fibroblasts and other cell types. (TIF) [file pcbi.1013027.s003.tif]

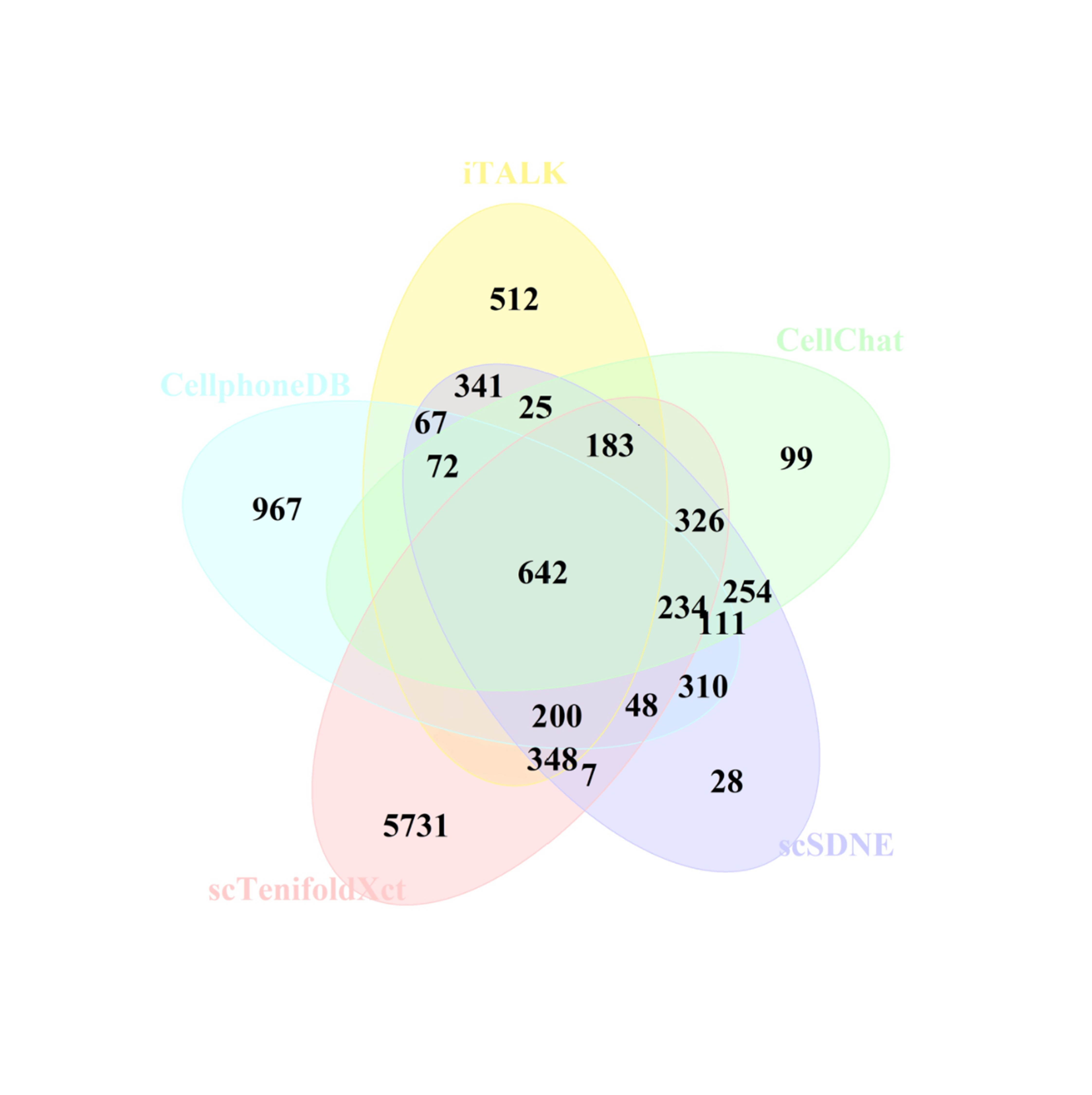

Supplement: S4 Fig — Overlap analysis of the LR database. (TIF) [file pcbi.1013027.s004.tif]
